# Use of Alkaline-Activated Energy Waste Raw Materials in Geopolymer Concrete

**DOI:** 10.3390/ma17102253

**Published:** 2024-05-10

**Authors:** Marta Nalewajko, Michał Bołtryk

**Affiliations:** Faculty of Civil Engineering and Environmental Sciences, Bialystok University of Technology, ul. Wiejska 45A, 15-351 Białystok, Poland; m.boltryk@pb.edu.pl

**Keywords:** lightweight geopolymer concrete, aluminosilicate artificial aggregate, fly ash, compressive strength of geopolymers, water absorption, bulk density

## Abstract

Silica fly ash, Certyd aggregate, and an alkaline solution were used to produce lightweight geopolymer concretes. The compressive strength, water absorption, and bulk density results, along with SEM photos showing the structure of the obtained composite, were obtained. Tests conducted on the specifications of lightweight geopolymer concretes have revealed significant chemical interactions between the ash aggregate and the geopolymer mortar, particularly when the coarse aggregate surface has been pre-treated with an alkaline solution. A statistical analysis of the experimental data, which investigated the influence of three key variables on the compressive strength, water absorption, and bulk density of lightweight geopolymer concrete (LBG), identified the following factors as having the most substantial impact: the quantity of alkali used, the curing temperature, and the concentration of alkali in the mixture. The optimal test series exhibited a commendable compressive strength of 20.14 megapascals (MPa), accompanied by a water absorption rate of 14.72%, and a bulk density of 1486.6 kg per cubic meter (kg/m³). These findings underscore the importance of alkali content, curing temperature, and alkali concentration in tailoring the properties of lightweight geopolymer concrete to meet specific performance requirements.

## 1. Introduction

A literature review shows that interest in fly ash-based geopolymer concrete first emerged in the 1980s and currently continues worldwide.

The technology for producing lightweight geopolymer concrete based on fly ash and aluminosilicate artificial aggregate was established with sustainable development in mind, aiming to reduce negative human impact on the environment. Sustainable development is based on proper resource management and caring for the natural environment. To achieve a sustainable state of development, designing specific consumer behaviors that ensure a balance between human needs and environmental possibilities is essential. The initial step in achieving these goals is to minimize the negative environmental impacts of activities within the industrial and construction sectors. This involves developing technologies for producing lightweight concrete from energy waste materials, while reducing the use of natural raw materials such as natural aggregates and environmentally harmful products like Portland cement.

In developing the research program, the elimination of Portland cement, previously the main component of concrete, was a priority. Studies have shown that producing one ton of cement releases 0.9 tons of CO_2_, accounting for 5% to 7% of global CO_2_ emissions [1,2,3]. Many studies have shown that geopolymer concrete is an alternative to cement-based concrete, and its synthesis is a process that consumes half the energy required for Portland cement production and releases 80% less carbon dioxide [4,5,6,7]. Geopolymer production can, therefore, significantly reduce the environmental burden by reducing gas emissions.

In addition to gas emissions, the use of certain industrial waste products, including fly ash, is also important. Fly ash is a highly dispersed material consisting of spherical particles represented by hollow aluminosilicate balls, with a diameter of 0.1–100 μm, responsible for the binding properties in alkali-activated lightweight concrete [8,9,10,11,12]. It allows for the elimination of the problem of waste stacking on specially prepared landfills, which has a significant impact on the environment.

Cement-free building materials were also obtained using ground perlite raw material using alkaline activation, as outlined in a 2022 article by Acar M.C., Çelik A.I., and Kayabaşı R., et al. [13]. Several tests were carried out, including compressive strength testing, which resulted in a level of 6–16 MPa. Cementless paste and mortar were produced based on the alkaline activation of raw perlite, and these proved suitable as building materials. However, the durability of the mixes decreased with increasing water content in both the paste and mortar. Most importantly, cement-free pastes and mortars can be produced in an environmentally friendly manner, with no high energy requirements and no greenhouse gas emissions [13]. 

Equally important is replacing natural aggregates, because of unwillingness to extract raw materials from protected natural environments, with recycled aggregates [14,15,16,17] or artificial aggregates made from industrial waste or by-products [18,19]. There are two techniques for producing artificial aggregates [20]: sintering [21,22], and cold bonding [23,24]. The aggregate used in the study was obtained by sintering, with simultaneous granulation, resulting in a lightweight, porous ceramic aggregate with high thermal insulation [25,26] and resistance to atmospheric and chemical factors, as well as fungi, insects, and rodents. In addition, it is odorless, highly crush-resistant, and has relatively low water absorption.

Replacing Portland cement in concrete with fly ash and natural aggregates with aluminosilicate artificial aggregate required research to demonstrate that the resulting product meets the requirements of the current norms and regulations and that it is comparable to traditional concrete. The fly ash was combined with artificial aggregate using an alkaline solution, resulting in a geopolymer, or artificial concrete. The production of geopolymers can thus lead to a significant reduction in environmental impacts by reducing emissions, utilizing certain waste products from the energy, mining, and metallurgical sectors, as well as by replacing natural raw materials with artificial fly ash aggregate, which can prove particularly useful in sustainable development-based construction [27].

In 2008, Obada Kayali conducted research using fly ash lightweight aggregates to produce high-strength concretes. Concrete produced using these aggregates was about 22% lighter and obtained strengths of 40–60 MPa. However, cement and natural aggregate were used in its production [28].

In 2015, Anja Terzic and Lato Pezo et al. [22] analyzed the impact of using granulated aggregates based on fly ash obtained by various processing techniques on the behavior of lightweight concretes. The experimental plan assumed the use of four lightweight artificial aggregates. The performance of lightweight concrete was compared with that of traditional concrete by testing, inter alia, compressive strength. An increase in concrete strength was observed due to increased ash fineness, which also affected the granules’ sintering time and the reduction of the sintering temperature. The 28- and 56-day-old lightweight concrete samples were characterized by properties meeting the requirements for traditional concretes, resulting in levels of 56–58 MPa. Cement, water, natural aggregate, and artificial aggregate were used to produce concrete in these studies [29].

The compressive strength test is essential in the context of the conducted analyses. Many publications have been released in which researchers focused on preparing cement-free composites based on alkali-activated fly ash and tested their basic properties. Eric Asa, Monisha Shrestha, Edmund Baffoe-Twum, and Bright Awuku researched cement-free composites based on fly ash and described their findings in an article in 2020. They focused on developing sustainable building materials using 100% calcium and potassium hydroxide (KOH) alkaline solution and examining the engineering properties of the resulting fly ash-based geopolymer concrete. They conducted laboratory tests to determine the mechanical properties, such as compressive and bending strength, of geopolymer concrete. In the study’s first phase, carbon nanotubes (CNT) were added to the mixture to determine their influence on the strength of geopolymer mortar. The results of the experiments showed that the mortar and concrete made entirely from fly ash require an alkaline solution to obtain strength properties comparable to those of Portland cement. However, it was found that increasing the amount of KOH generates a significant amount of heat, causing the concrete to set too quickly. The study also showed that adding CNT to the mixture makes the geopolymer concrete harder than traditional concrete without CNT [30].

In the same year, Zijian Su, Wei Hou, and others conducted research in which they synthesized a foamed geopolymer from fly ash to produce geopolymer-based lightweight concrete (GLWC). The fly ash was activated with a sodium silicate solution, and aluminum powder was used as a foaming agent. The synthesized mortars were cured at 40 °C for 28 days, resulting in a product with a bulk density ranging from 600 to 1600 kg/m^3^. The results showed that GLWC had higher mechanical strength than commercial cellular concrete and achieved 80–90% of its 28-day strength after 7 days of curing. In addition, for densities ranging from 600 to 1200 kg/m^3^, the thermal conductivity decreased from 0.70 to 0.22 W/m·K, which is significantly better than the result for its counterpart, ordinary Portland cement (OPC) concrete [31].

The article presented geopolymer concrete produced using various waste additives, different concentrations of alkaline solution, and a range of curing conditions involving varying times and temperatures. The resulting conclusion emphasized the significantly improved physico-mechanical properties displayed by geopolymer composites when compared to those of traditional Portland cement-based concretes. The research results allowed for the granting of a patent [32]. Based on available domestic and international literature sources, a decision was made to conduct study lightweight concretes formulated by incorporating alkali-activated fly ash, supplemented by the inclusion of aluminosilicate artificial aggregates. It is worth noting that the literature review did not yield any information regarding waste additives in the form of such aggregates. Previous studies exclusively employed natural aggregates, with artificial aggregates taking the form of fibers, chips, sawdust, or foam. The literature analysis unequivocally confirmed that, as of the present date, no research has been undertaken in the field of integrating aluminosilicate artificial aggregates into geopolymer concretes. Based on research conducted by K. Kalinowska-Wichrowska et al. 2022 [29], who proposed a solution to improve the adhesion of geopolymer grout to coarse artificial aggregate with high porosity by impregnating the 4–8 mm fraction of the aggregate with an alkali solution, it was decided to employ the surface impregnation of coarse aggregate. 

A hypothesis was put forward that it is possible to monolithically bind the alkali-activated precursor with artificial lightweight aggregate, which would improve the properties of lightweight concrete and eliminate the need for Portland cement. The aim was to develop a recipe for cement-free composites based on raw waste energy materials activated by alkalis and to investigate their properties. The research completely eliminated the use of Portland cement and natural aggregates, relying solely on waste raw materials and artificial aggregates. The existing literature on geopolymer concretes mostly focuses on formulations that only partially replace natural aggregates or Portland cement.

The article presents the materials used to produce the lightweight geopolymer concretes studied. It also describes the method of producing these specific concretes and includes basic test results for compressive strength, water absorption, and bulk density, along with SEM photos and XRD test results. The article concludes with a discussion and final conclusions.

## 2. Materials and Methods

### 2.1. Materials

For the production of lightweight concrete based on alkaline-activated waste raw materials, the following were used:

- 1 part fly ash;
- 0.5 part alkaline solution;
- A 2, 4, 6 moles/dm^3^ concentration of alkaline solution in the amount of 100, 200 or 300 [kg/m^3^] (OLTCHIM – Romania; ANSER – Poland);
- A 2.5 ratio of sodium silicate to sodium hydroxide;
- Artificial aggregate fractions 0–2 mm, 1–4 mm, and 4–9 mm (where 50% of the amount of aggregate was 4–9 mm aggregate, and the fractions 0–2 mm and 1–4 mm each constituted 25% of the amount of aggregate).

#### 2.1.1. Silica Fly Ash

**Figure 1:**
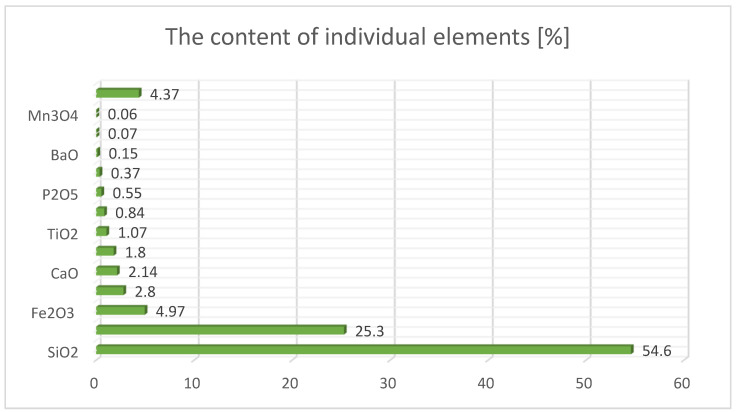
Content of basic elements in silica fly ash.

Lightweight concretes based on alkali-activated waste materials were prepared using fly ash, consisting of spherical, vitrified fine grains from coal combustion produced by a power plant in Ostrołęka. Figure 1 presents the content of the basic elements in the silica fly ash used in the study. 

#### 2.1.2. Fly Ash Aggregate

Artificial aggregates are characterized by low bulk density, very high porosity (20–50%), and as a result, high water absorption and low crushing strength, depending on the fraction. Regarding their oxide composition, most of them contain large amounts of silica and aluminum oxide, which can enter into a polymerization reaction with alkalis, such as sodium hydroxide. 

**Figure 2:**
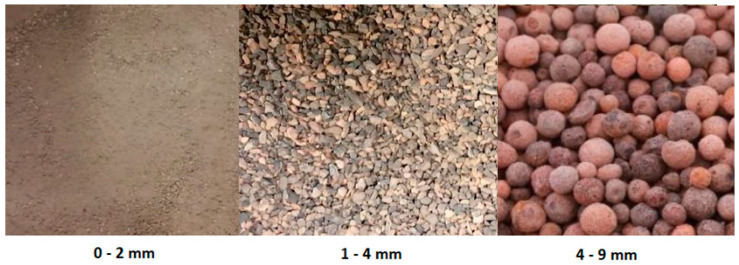
Certyd-type lightweight ash aggregate, with particle sizes of 0–2 mm, 1–4 mm, and 4–9 mm.

Certyd is a lightweight waste aggerate made from fly ash, produced by the LSA Aggregate Production Plant, in fractions of 0–2 mm, 1–4 mm, and 4–9 mm. The coarse aggregate was surface-impregnated with an alkaline solution with an appropriate NaOH concentration before being added to the geopolymer mixture to reduce porosity and increase adhesion to the geopolymer paste. After hardening, the impregnated aggregate was evaluated for water absorption and crushing strength. The results showed that the impregnated coarse aggregate exhibited significantly reduced absorbability when compared to the non-impregnated aggregate. The crushing index, which is a measurement of crushing strength for high-class concrete aggregates, was 27.3% for the non-impregnated coarse aggregate. However, the impregnated coarse aggregate achieved a crushing index in the range of 14–15%, which falls within the acceptable limit for high-class concrete aggregates. Furthermore, the crushing index decreased with higher concentrations of the activator, indicating that impregnation with an alkali solution improves the suitability of coarse ash-pore aggregate for high-class concrete applications. They also compared the compressive strength of composites containing impregnated aggregate with composites containing non-impregnated aggregate, noting that impregnation of the aggregate increases the compressive strength of concrete by almost double [29]. Table 1 presents the properties of Certyd aggregate for each fraction, and Figure 2 shows the three fractions of the aggregate used in the research.

An ash-porous aggregate gaining popularity for use in Poland is Certyd, made of sintering fly ash from electrostatic precipitators and an ash-slag mixture from the wet discharge of combustion waste from the hard coal combustion process in fine coal boilers. The aggregate production technology is based on two processes: drying and firing, with the sintering of ash granulates using a rotary kiln operating in a co-current media system. Air is supplied in a radial system through the armor. The sintering process is carried out without external fuel, using only the combustion heat emitted in the production process from the remains of carbon contained in the ash, ranging from 5.5% to 19% of the coal weight.

In the laboratory of the Faculty of Civil Engineering and Environmental Sciences of Bialystok University of Technology, the aggregate was also tested for the percentage content of silica and aluminum, obtaining the results provided in Table 2.

#### 2.1.3. Alkaline Activator

The alkaline activators of various concentrations ranging from 2 to 6 mol/dm^3^ were used for the research. The sodium hydroxide solution with water glass, referred to as the alkaline solution or activator in the article, was prepared from solid sodium hydroxide flakes from OLTCHIM, which were dissolved in distilled water to obtain the sodium hydroxide solution by the exothermic reaction. To cool the solution to room temperature, it was left for 24 h and then mixed with sodium silicate solution. The mass ratio of sodium silicate solution to sodium hydroxide solution (Na_2_SiO_2_/NaOH) was constant and equal to 2.5. The activator was the R-145 water glass solution from ANSER. Table 3 shows the mass ratio of solid NaOH to distilled water.

### 2.2. Methods

Below is presented the method of making a geopolymer mixture based on fly ash, alkaline solution, and fly ash aggregate, which was then placed in molds, curing at temperatures consistent with the experimental plan, cured for 28 days, and then tested for compressive strength, water absorption, and bulk density; the results from SEM microscopy and the XRD tests are also presented.

The research aimed to investigate the influence of NaOH concentration, the amount of the activator, and the curing temperature on the properties of lightweight alkali-activated concrete made from waste materials. The following explanatory variables were adopted in the experiment:

X_1_—the amount of the activator: 100, 200, 300 [kg/m^3^]; 

X_2_—the concentration of the alkaline solution: 2, 4, 6 [mol/dm^3^];

X_3_—the curing temperature: 25 °C, 65 °C, 105 °C. 

Table 4 shows the range of variability of the factors under consideration. The geopolymer mixture based on waste materials in the form of alkali-activated silica fly ash and fly ash aggregate was prepared according to the experiment plan, assuming that in all series, the fly ash aggregate of the 4–9 mm fraction will be surface impregnated with the alkali solution. 

Table 5 shows the selection of the geopolymer mixture composition. The alkaline activators of various concentrations ranging from 2 to 6 mol/dm^3^ were used for the research. In the preliminary research, it was established that in the case of the aluminosilicate artificial aggregate, the molality of 2–6 is sufficient. Such a range was also dictated by the reduced consumption of alkali in lightweight geopolymer concretes, which translates into economic and environmental benefits.

Moreover, alkaline activation can occur at low temperatures, which means that making building materials using alkaline activation is more energy efficient than traditional cement making methods. According to the literature analysis, the polycondensation reaction is not a spontaneous reaction and requires an external energy supply. It initiates after increasing the temperature above 35 °C. It is also assumed that the polycondensation reactions of geopolymers occur at room temperature, but temperatures of 60–105 °C are considered optimal [38]. The analysis dictated the recipe selection, the results of the preliminary research, and the economic considerations.

Before the impregnation of the aggregate, tests were carried out on the absorption of the given aggregate and the crushing strength of the non-impregnated and surface-impregnated aggregate. Due to minor differences in the mass increase of the coarse aggregate, it was decided to impregnate the surface of the aggregate for 10 s at a later stage of the research. In addition, it was noticed that this increase was about 30% of the weight of the coarse aggregate; therefore, in the following research stage, the coarse aggregate was surface impregnated in an alkaline solution corresponding to 30% of the weight of the coarse aggregate. In the case of the crushing strength test, the non-impregnated aggregate of a 4–8 mm fraction was tested in the first stage, obtaining a crushing strength of 25.13%. In the next stage, the impregnated aggregate was tested with an alkaline solution, obtaining a crushing index in the range of 14.39–14.78% (depending on the concentration of NaOH in the solution). Therefore, the results showed that the ash aggregate impregnated with an alkaline solution is suitable for use in high-grade concrete.

**Figure 3:**
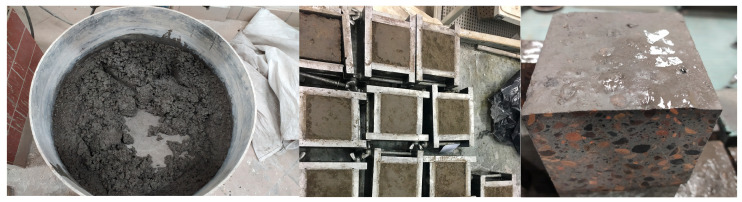
Phases of creating cementless composites.

The geopolymer concrete mix preparation consisted of measuring the appropriate amount of each component. First, the aggregate of the 4–9 mm fraction was surface impregnated with an alkaline solution of the appropriate concentration for 10 s, then sieved, getting rid of the excess solution, and weighed. Next, cement and the ash aggregate of 0–2 mm and 1–4 mm fractions were poured into the mixer drum. All the ingredients were mixed for 60 s, the mixer was stopped, and then the ingredients were mixed by hand. The ingredients were mixed again for 60 s; after stopping, the drum was filled with an impregnated aggregate of the 4–9 mm fraction and an alkaline solution of the appropriate concentration. The entire content was mixed for 60 s. The mixer was stopped for the fourth time; the ingredients were mixed manually in order to separate the ingredients from the drum walls, and the device was turned on again for 60 s. The last step was repeated twice. The entire mixing process took 5 min. The ready mix was partly placed in steel molds, previously covered with grease in order to protect them against adhering to the mix, with dimensions of 10 cm × 10 cm × 10 cm, corresponding to the PN-EN 12390-1 standard [39]. The compaction of the molds ensured vibro-pressing and vibration for 30 s, followed by vibro-pressing for another 30 s. Figure 3 below shows the phases of creating cementless composites.

The samples were left to air dry for 24 h, and then they were placed in a dryer heated to the appropriate temperature for another 24 h. Next, the samples were demolded and placed by the water for 28 days from the molding date. After this period, they were tested for compressive strength, water absorption, bulk density, and capillary rise. 

## 3. Results and Discussion

### 3.1. Compressive Strength Test

**Figure 4:**
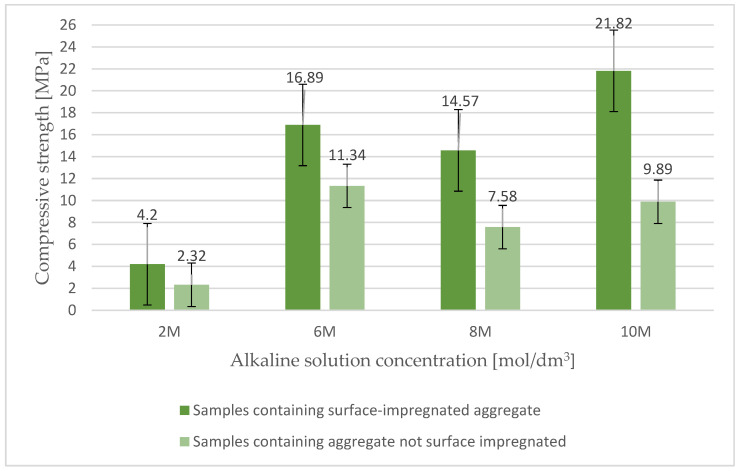
The results of the compressive strength of lightweight geopolymer concrete based on surface-impregnated Certyd and fly ash.

The influence of coarse ash aggregate surface impregnation on the properties of lightweight concrete based on alkali-activated waste materials was investigated. Figure 4 presents the results for the compressive strength of lightweight geopolymer concrete based on surface-impregnated artificial aggregate and fly ash. The analysis of the test results, aimed at examining the effect of the activator concentration on the properties of geopolymer concrete, indicated that the surface impregnation of coarse aggregate with the activator causes an almost two-fold increase in compressive strength. These premises required that the main tests were carried out using coarse ash-porous aggregate impregnated on the surface. The surface impregnation of the lightweight aggregate yielded a mixture of appropriate workability and consistency, thus improving the properties of ready-made lightweight geopolymer composites based on waste energy raw materials activated with alkali.

The initial analysis focused on the influence of the concentration of the alkaline solution, the amount of the activator, and the curing temperature on the compressive strength, water absorption, and bulk density of the geopolymer composites. One-dimensional significance tests were conducted to obtain average boundary values, which allowed for the determination of the explanatory influence of the indicated variables on strength, water absorption, and bulk density.

The results of the compressive strength tests of geopolymer lightweight concrete were subjected to preliminary analysis, and basic statistical measures were determined and presented in Table 6. The differences between strength values from the individual stages of preliminary testing were minor; therefore, in the analysis conducted, they were deemed insignificant.

Based on the results presented in Table 6, it can be concluded that the highest compressive strength was obtained with an alkaline solution of 300 kg/m^3^, a concentration of 6 mol/dm^3^, and a curing temperature of 105 °C. The average compressive strength was 26.92 MPa, with a relatively low variance of 0.312 MPa^2^. The variability of the results with these parameters was very low, at approximately 2.07%. The second-best result was obtained with an alkaline solution of 200 kg/m^3^, a concentration of 6 mol/dm^3^, and a curing temperature of 65 °C. In this case, the average compressive strength was 22.86 MPa, with a variance of 0.508 MPa^2^. The variability of the results with these parameters was similarly low, at approximately 3.12%. The samples with the lowest compressive strength had an alkaline solution of 100 kg/m^3^, a concentration of 2 mol/dm^3^, and a curing temperature of 25 °C. The average compressive strength was only 0.33 MPa, with a variance of 0.0083 MPa^2^. The variability of the results with these parameters was very high, at approximately 27.52%. The coefficient of variation Vi for each sample, which characterizes the relative variation of the observed variable Y, ranged from 2.07% to 27.52%.

After calculating the regression coefficients, the following equation was obtained to describe the relationship between compressive strength and the indicated factors (1):

(1) $$Y_{w}=-16.8385+0.0439\;*\;X_{1}+1.9453\;*\;X_{2}+0.1354\;*\;X_{3}$$

where Y is dependent variable, and X_1_, X_2_, and X_3_ are the independent variables.

The interpretation of the coefficients of the obtained model leads to the conclusion that an increase in the amount of the activator by 1 kg/m^3^ results in an increase in compressive strength of alkali-activated lightweight concrete by 0.04 MPa, while an increase in the activator concentration by 1 mol/dm^3^ leads to an increase in compressive strength of 1.95 MPa. Additionally, an increase in the curing temperature by 1 °C also results in an increase in the compressive strength of this geopolymer by approximately 0.14 MPa.

The results in Table 7 indicate that the highest compressive strengths of lightweight concrete made from alkaline-activated waste materials were obtained in series 8. The mean compressive strength was 26.92, and the standard deviation was 0.5586. These results are based on a small random sample of *n* = 5. The results of random samples are the basis for statistical inference, which may include estimating unknown parameter levels (e.g., expected values) in the general population. Of course, their statistical estimation is performed under uncertainty, with a declared probability. Point estimation (which is highly dangerous) or interval estimation can be made, which covers the estimated parameter at the declared level of confidence, and is much safer. In this study, a Neyman confidence interval was determined for the expected value of the variable being tested, based on the results obtained in series 8, assuming that the estimator of the estimated parameter is the arithmetic mean obtained in this random sample and with a confidence level of α = 0.05 (2):

(2) $$P\left(26.92-0.78<m<26.92+0.78\right)=0.95$$

where P is the Neyman confidence interval, and m is the expected value.

$$P\left(26.14<m<27.70\right)=0.95$$

The determined interval is one that covers the expected value of the variable being tested at the declared confidence level. Therefore, the result obtained indicates that with 95% confidence, the mean compressive strength of lightweight concrete made from alkaline-activated waste materials (in the general population) is between 26.14 MPa and 27.70 MPa. An essential part of the conducted analysis is to indicate the absolute and relative precision of the estimation, which is necessary for this type of research. The absolute precision of estimation is ∆=0.78.

The relative precision of estimation, expressed in percentages, allows us to decide whether the inference can be considered statistically safe. The relative precision of estimation is δ=2.90%, and it is lower than 5%, meaning that the inference can be considered entirely safe. Importantly, achieving such a favorable result with such a small random sample is particularly satisfying. Increasing the number of units in random samples leads to a significant increase in estimation precision due to the decrease in the range of random endpoints of the confidence intervals.

Similar research was published by Wichrwska-Kalinowska K., Pawluczuk E., and others in 2022 [29], who produced lightweight geopolymer concretes based on fly ash or a mixture of fly ash with a slag and lightweight aggregates of the Certyd type. They tested compressive strength, water absorption, apparent density and thermal conductivity. In the case of compressive strength, they obtained results from 2.54 MPa to 16.38 MPa (average strength, 7.23 MPa), which are lower than those obtained in the tests presented in another given article, where the compressive strength was in the range of 0.33 MPa up to 26.92 MPa (average strength 8.52 MPa). The difference in the highest compressive strengths obtained for lightweight geopolymer concretes was 10.54 MPa. 

A higher strength of 41 MPa was achieved by Ge, W.; Chen, J.; Min, F.; Song, S.; Liu, H. [5] for metakaolin-based geopolymers synthesized with quartz activated with an alkali dose of 5%.

Most publications focus on the production of lightweight geopolymer concretes based on natural aggregates, in the case of which the compressive strengths are usually higher (up to 60 MPa [28]) than those obtained in the production of geopolymers based on ash aggregates, due to the high porosity of the given aggregate.

### 3.2. Water Absorption Tests

The results for the water absorption tests of geopolymer lightweight concrete were subjected to preliminary analysis, and basic statistical measures were determined and are presented in Table 7. 

**Table 7:** The results of water absorption of the geopolymers.

Activator [kg/m^3^]	Curing Temperature [°C]	Concentration of Alkaline Solution [mol/dm^3^]	Series No.	Arithmetic Mean of Water Absorption [%]	Standard Deviation	Variance	Coefficient of Variation [%]
Activator 100	25	2	(1)	20.07	0.4518	0.2041	2.2511
		6	(5)	21.65	0.5218	0.2722	2.4104
	65	4	(11)	16.49	0.1578	0.0249	0.9569
	105	2	(2)	17.37	0.2545	0.0648	1.4653
		6	(6)	20.66	0.2541	0.0646	1.2298
Activator 200	25	4	(9)	21.64	0.3933	0.1547	1.8172
	65	2	(13)	15.17	0.2803	0.0786	1.8484
		6	(14)	16.56	0.3662	0.1341	2.2114
	105	4	(10)	14.37	0.1031	0.0106	0.7177
Activator 300	25	2	(3)	14.72	0.2975	0.0885	2.0208
		6	(7)	21.53	0.2671	0.0713	1.2403
	65	4	(12)	16.43	0.1627	0.0265	0.9902
	105	2	(4)	14.87	0.3501	0.1226	2.3542
		6	(8)	14.19	0.2672	0.0714	1.8829

Based on the results in Table 7, it can be concluded that the lowest water absorption was achieved with an activator amount of 300 kg/m^3^, a concentration of 6 mol/dm^3^, and a heating temperature of 105 °C. The average water absorption of the tested samples was 14.19%, with a very low variance of 0.07%^2^. The diversity of results with these parameters was very low, approximately 1.88%. The second most favorable result was obtained with an activator amount of 200 kg/m^3^, a concentration of 4 mol/dm^3^, and a heating temperature of 105 °C. The average water absorption in this case was 14.37%, with a variance of 0.01%^2^. The diversity of results with these parameters was very low, approximately 0.72%. The highest absorbability was demonstrated by samples with an activator amount of 100 kg/m^3^, a concentration of 6 mol/dm^3^, and a heating temperature of 25 °C. The average water absorption was 21.65%, with a variance of 0.27%^2^. The variation in results with these parameters was around 2.41%, which may indicate the reliability of the results obtained in individual samples. The values of the coefficient of variation Vi for each sample, characterizing the measure of the relative variability of the observed value of the Y variable, ranged from 0.96% to 14.25%.

The model coefficients were calculated using the single least squares method. After calculating the values of the regression coefficients, the following form of the equation describing the dependence of compressive strength on the indicated factors was obtained (1):

$$Y_{w}=20.92195-0.01448\;*\;x_{1}+0.61915\;*\;x_{2}-0.04540\;*\;x_{3}$$

Interpreting the coefficients of the obtained model, it can be concluded that an increase in the amount of the activator by 1 kg/m^3^ causes a decrease in the water absorption of lightweight concretes based on alkaline-activated waste raw materials by approximately 0.01%, while an increase of 1 mol/dm^3^ in the concentration of the activator causes an increase in the water absorption of concrete by approx. 0.62%, while an increase in the maturing temperature by 1 °C causes a decrease in the water absorption of this geopolymer by approx. 0.05%.

The results in Table 8 clearly indicate the lowest absorption rates for lightweight concrete based on alkaline-activated waste raw materials in series 7, where the average water absorption is 14.19% and the standard deviation is 0.27%. The results of random sample observations, which constitute the basis for statistical inference, allow for, among other things, the estimation of an unknown level of a parameter in the general population. At the declared level of probability, an interval estimate of the expected water absorption of geopolymers was performed. The results obtained in series 8 were used as the basis for estimation, assuming that the estimator of the estimated parameter is the arithmetic mean obtained in this random sample, and the confidence level is α = 0.05 (2):

$$P\left(14.19-0.37<m<14.19+0.37\right)=0.95$$

$$P\left(13.82<m<14.56\right)=0.95$$

The designated interval covers the expected value of the tested variable at the declared confidence level. The obtained result means that, with 95% certainty, the average water absorption of lightweight concretes based on alkaline-activated waste raw materials (in the general population) ranges from 13.82% to 14.56%. The absolute precision of the estimate is ∆ = 0.37 [%]. The relative precision of the estimation, expressed as a percentage, allows for the assessment of whether the inference can be considered statistically safe. The level of relative estimation precision is δ = 2.61%, and it lower than 5%, which means that the inference can be considered completely safe.

The water absorption of lightweight geopolymer concretes based on fly ash and Certyd aggregate was also presented in the article by Kalinowska-Wichrowska K., Pawluczuk E., and others in 2022 [29], where it was in the range of 14.2% to 25.6% (with an average of 28.3%). In the tests presented in the given article, the water absorption values ranged from 14.19% to 21.65% (with an average of 17.55%). Also in this case, a lower water absorption of lightweight geopolymer concretes was achieved.

Significantly higher water absorption rates, ranging from 12% to 35%, were also observed in the synthesized GLWCs presented in the article by Zijian Su, Wei Hou, and others [31].

In the case of tests carried out on lightweight geopolymer concretes based on natural aggregates, much lower water absorption is achieved compared with that observed for those based on ash aggregates, which is related to the porous structure of artificial aggregates.

### 3.3. Bulk Density Tests

The results of bulk density tests of geopolymer lightweight concrete were subjected to preliminary analysis, and basic statistical measurements were determined and are presented in Table 8. 

**Table 8:** The bulk density results for the geopolymers.

Activator [kg/m^3^]	Curing Temperature [°C]	Concentration of Alkaline Solution [mol/dm^3^]	Series No.	Arithmetic Mean of Bulk Density [g/dm^3^]	Standard Deviation	Variance	Coefficient of Variation [%]
Activator 100	25	2	(1)	1415.24	10.2473	105.008	0.7241
		6	(5)	1355.76	15.5425	241.568	1.1464
	65	4	(11)	1673.76	22.8479	522.028	1.3651
	105	2	(2)	1565.60	36.3995	1324.925	2.325
		6	(6)	1418.44	13.9402	194.328	0.9828
Activator 200	25	4	(9)	1387.60	9.7026	94.140	0.6992
	65	2	(13)	1705.80	18.1611	329.825	1.0647
		6	(14)	1592.76	37.1242	1378.208	2.3308
	105	4	(10)	1709.16	16.8389	283.548	0.9852
Activator 300	25	2	(3)	1600.28	39.5645	1565.352	2.4724
		6	(7)	1457.76	45.5547	2075.228	3.125
	65	4	(12)	1653.30	12.8092	164.075	0.7748
	105	2	(4)	1756.68	18.3001	334.892	1.0417
		6	(8)	1704.72	13.2322	175.092	0.7762

Based on the results in Table 8, it can be concluded that the lowest density was obtained with an activator amount of 100 kg/m^3^, a concentration of 6 mol/dm^3^, and a heating temperature of 25 °C. The average density of the tested samples was 1355.76 g/dm^3^, with a variance of 241.5680 (g/dm^3^)^2^. The diversity of results with these parameters was very low, approx. 1.15%. The second relatively favorable result was obtained with an activator amount of 100 kg/m^3^, a concentration of 6 mol/dm^3^, and a heating temperature of 105 °C. 

The medium density in this case is 1387.60 g/dm^3^, with a variance of 94.1400 (g/dm^3^)^2^. The variation in the results with these parameters was very low, approximately 0.7%. The highest density was obtained in samples with an activator amount of 300 kg/m^3^, a concentration of 2 mol/dm^3^, and a heating temperature of 105 °C. The average bulk density obtained was as high as 1756.68 g/dm^3^, with a variance of 334.8920 (g/dm^3^)^2^. The variation in the results with these parameters was not high and was approximately 1.0417%, which may indicate the reliability of the results obtained in the individual samples. The values of the coefficient of variation Vi for each sample, characterizing the measure of the relative variability of the observed value of the variable Y, range from 0.7% to 5.4%.

The regression function, determined using a single least squares method, describing the dependence of bulk density on the amount of the activator, its concentration, and its annealing temperature, takes the form (1):

$$Y_{w}=1366.187+0.784\;*\;x_{1}-23.708\;*\;x_{2}+2.245\;*\;x_{3}$$

The interpretation of the coefficients of the obtained model leads to the conclusion that an increase in the amount of the activator by 1 kg/m^3^ causes an increase in the bulk density of lightweight concretes based on alkaline-activated waste raw materials by approximately 0.078 g/dm^3^, while an increase in the activator concentration by 1 mol/dm^3^ causes a decrease in concrete density by approx. 23.71 g/dm^3^. However, an increase in the ripening temperature by 1 °C results in an increase in the density of this geopolymer by approximately 2.245 g/dm^3^.

The results of random sample observations, which constitute the basis for statistical inference, allow for, among other things, the estimation of an unknown level of a parameter in the general population. At the declared level of probability, an interval estimate of the expected value of the bulk density of geopolymers was made. The results obtained in series 5 were used as the basis for estimation, assuming that the estimator of the estimated parameter is the arithmetic mean obtained in this random sample, and the confidence level is α = 0.05 (2):

$$P\left(1355.76-21.57<m<1355.76+21.57\right)=0.95$$

$$P\left(1334.19<m<1377.33\right)=0.95$$

The designated interval is one that covers the expected value of the tested variable at the declared confidence level. The obtained result means that, with 95% certainty, the average density of lightweight concretes based on alkaline-activated waste raw materials (in the general population) ranges from 1 334.19 g/dm^3^ to 1 377.33 g/dm^3^.

The absolute precision of estimation is ∆=21.57 [g/dm^3^]. In turn, the relative precision of estimation, equal to δ=1.59%, allows us to assess whether such inferences can be considered statistically safe.

The bulk density of lightweight geopolymer concretes based on fly ash and Certyd aggregate is also available in the article by Kalinowska-Wichrowska K., Pawluczuk E., and others, published in 2022 [29], which shows that it ranges from 1210 g/dm^3^ to 1540 g/dm^3^ (average 1530 g/dm^3^). In the research presented in the article, the obtained bulk density reanged from 1.356 g/dm^3^ to 1.757 g/dm^3^ (average 1.571 g/dm^3^). Considering the highest bulk densities obtained in the research presented in the article, the obtained bulk density was 218 g/dm^3^ higher.

### 3.4. SEM Photos and XRD Test

**Figure 5:**
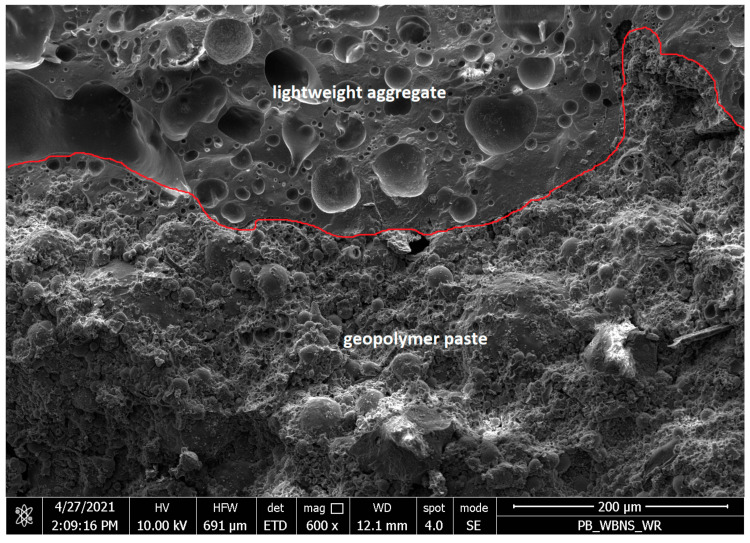
Samples of lightweight concrete based on fly as and activated alkaline aluminosilicate artificial aggregate, under approximately 600× mag, with the contact zone between the lightweight aggregate and the geopolymer paste marked in red.

**Figure 6:**
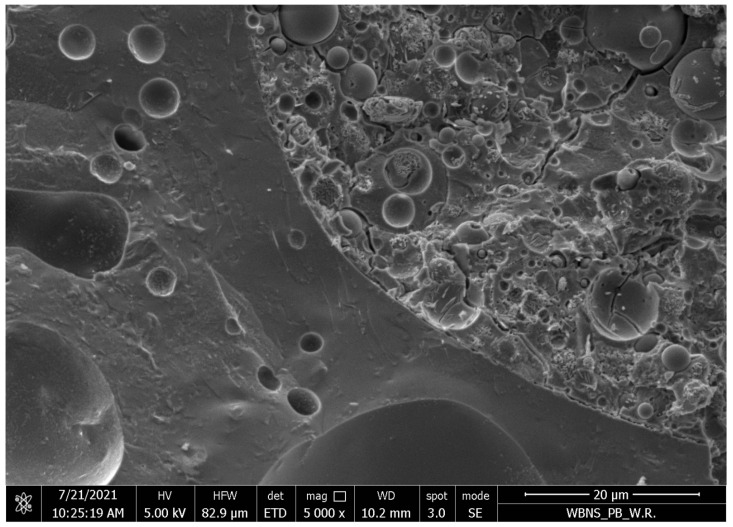
Samples of lightweight concrete based on fly ash, activated with sodium hydroxide solution aluminosilicate artificial aggregate, under approximately 5000× mag, with the contact zone between the lightweight aggregate and the geopolymer paste.

Lightweight concrete samples based on the fly ash in an amount of 600 kg/m^3^, aluminosilicate artificial aggregate activated by a 6 mol/dm^3^ alkali solution, and ripened at a temperature of 105 °C, were also examined under a scanning electron microscope and showed a fine-grained structure with a small number of micro and macro cracks, as shown in Figure 5. The interfacial transition zone between the GP and the aggregate was determined to be very good. The majority of the fly ash had reacted. Figure 6 shows samples produced with sodium hydroxide instead of an alkaline solution.

The best microstructure was achieved in lightweight geopolymer concretes containing 400–600 kg/m^3^ of fly ash, corresponding to 200–300 kg/m^3^ of an alkali solution with a concentration of 4 mol/dm^3^ and a maturation temperature of 105 °C. Then, we obtain a monolithic combination of polycondensation products with coarse aggregate, with a small number of pores, where a significant amount of fly ash particles undergo polymerization, creating a fine-grained structure of silica and aluminum oxide copolymers and stabilizing metal cations, mainly sodium and calcium, and bound water.

The analysis of the results presented in Table 9 for the semi-quantitative tests of the composition of the areas of the aggregate–geopolymer grout contact zone in sample of series 8, which was made by the Institute of Construction Technology, showed that:

- There was a higher overall porosity of the tested sample based on the higher concentration of carbon that comes from the resin filling the air pores;
- Locally, in the contact zone (0–80 μm from the edge of the aggregate) of the tested sample, the pore content was higher;
- There was an increased concentration of sodium ions in the area of the contact zone (0–80 μm from the edge of the aggregate) of the tested sample compared to greater distances from the aggregate boundary;
- In the tested sample, the maximum concentration of silicon and aluminum ions was observed in zone 3 (160–240 μm from the edge of the aggregate).

**Table 9:** Results of semi-quantitative compositional analyses of sample 8.

Distance from the Edge of the Aggregate [μm]	0–80	80–160	160–240	240–320	320–400	400–480	480–560	560–640	640–720	720–800
	Concentration [% of Mass]									
C	60.86	58.65	57.16	57.96	61.43	67.01	64.76	64.20	67.42	64.36
Si	19.99	21.99	23.08	22.60	20.82	17.96	18.56	18.97	16.82	18.68
Al	7.51	8.63	9.13	9.07	8.30	7.24	7.74	7.64	7.13	7.79
Na	5.34	5.15	4.87	4.79	4.21	3.80	3.61	3.84	3.63	4.38
Fe	3.92	3.16	3.28	3.43	3.03	2.25	3.02	3.22	2.94	3.13
Ca	2.38	2.41	2.48	2.15	2.20	1.74	2.31	2.13	2.06	1.66

## 4. Conclusions

Based on the results of the conducted research and the statistical analysis, the following conclusions are presented:

- Tests on the properties of lightweight geopolymer concretes have shown that there is a monolithic connection between the ash aggregate (after impregnating the aggregate surface of the 4–9 mm fraction with an alkaline solution) and the geopolymer mortar.
- The highest average compressive strength was obtained for series 8, characterized by a concentration of alkaline solution of 6 mol/dm^3^ and a curing temperature of 105 °C, with an activator dosage of 300 kg/dm^3^. The lowest values were observed for series 1. The observation suggests that a low concentration of alkaline solution, an insufficiently high curing temperature, and the amount of activator negatively affect the compressive strength of the geopolymer concrete.
- Statistical inference methods, based on the results of a random sample, allowed for an interval estimation of the parameter, which is the mean compressive strength. The results from the most favorable series of tests determined that with 95% confidence, the mean compressive strength of lightweight geopolymer concrete lies in the range of 26.14 MPa to 27.70 MPa. Therefore, the inference can be considered completely safe, as confirmed by the relative precision of estimation, which is 2.9% (lower than 5%).
- The best microstructure was obtained by lightweight geopolymer concretes containing 400–600 kg/m^3^ of fly ash, corresponding to 200–300 kg/m^3^ of an alkali solution with a concentration of 4 mol/dm^3^ and a maturation temperature of 105 °C. A monolithic combination of polycondensation products with coarse aggregate, with a small number of pores, is then obtained, where a significant number of fly ash particles undergo polymerization, creating a fine-grained structure of silica and aluminum oxide copolymers and stabilizing metal cations, mainly sodium and calcium, and bound water.
- Comparing the obtained test results for lightweight geopolymer concretes based on fly ash and fly ash aggregate with those published by other researchers, slightly better compressive strength and water absorption and slightly worse bulk density were obtained.
- The obtained results and the literature analysis revealed that a geopolymer composite based on raw waste materials, activated with alkali, with great compressive strength of 26 MPa, was obtained. Furthermore, higher strengths were obtained by researchers in the case of the partial use of natural aggregates and cement.

## 5. Patents

Bołtryk Michał, Nalewajko Marta, Nietupski Adam, the method of producing lightweight geopolymer concrete on aluminosilicate artificial aggregate (invention), application confirmed, application number: P.442271, date of application: 14 September 2022.

## Figures and Tables

**Table 1:** Properties of Certyd fly ash aggregate.

Technical Parameters	Standards/Procedures	0/2	1/4	4/9
Bulk density [kg/m^3^]	PN-EN 1097-3:2000 [33]	900 ± 10%	900 ± 10%	700 ± 10%
Thermal conductivity [W/m·K]	PN-EN 12667:2002 [34]	0.18	0.16	0.14
Chloride content [%]	PN-EN 1744-1:2010 [35]	0.00	0.00	0.00
Sulfate content soluble in acid [%]		0.25	0.25	0.25
Total sulfur content in terms of S [%]		0.32	0.32	0.32
Alkali-reactivity (rapid method)	PN-B 06714-46:1987 [36]	0.00	0.00	0.00
Radioactivity [Bq/kg]	Instruction ITB nr 455/2010 [37]	f1 < 1.2 f2< 240	f1 < 1.2 f2< 240	f1 < 1.2 f2< 240

**Table 2:** The content of silica and aluminum oxides on the surface of the Certyd aggregate.

Fraction	0–2 mm	1–4 mm	4–9 mm
	%	%	%
Al_2_O_3_	13.36	10.09	10.44
SiO_2_	10.83	8.44	8.11

**Table 3:** The ratio of the mass of solid NaOH to distilled water.

Concentration of Alkaline Solution [mol/dm^3^]	Weight of NaOH in 1 kg of Solution [g]	Mass of Water in 1 kg of Solution [g]
2	80	920
4	140	860
6	239	761
8	260	740
10	314	686

**Table 4:** The range of variability of the factors under consideration.

No.	X_1_	X_2_	X_3_	Amount of Alkaline Solution [kg/m^3^]	Concentration of Alkaline Solution [mol/dm^3^]	Curing Temperature [°C]
1	−1	−1	−1	100	2	25
2	+1	−1	−1	100	2	105
3	−1	+1	−1	300	2	25
4	+1	+1	−1	300	2	105
5	−1	−1	+1	100	6	25
6	+1	−1	+1	100	6	105
7	−1	+1	+1	300	6	25
8	+1	+1	+1	300	6	105
9	−1	0	0	200	4	25
10	+1	0	0	200	4	105
11	0	−1	0	100	4	65
12	0	+1	0	300	4	65
13	0	0	−1	200	2	65
14	0	0	+1	200	6	65

**Table 5:** Selection of the geopolymer mix composition.

No.	Fly Ash	Activator	Activator Used for Impregnation	Aggregate (Certyd)			Curing Temperature	Concentration of Alkaline Solution
				0–2 mm	1–4 mm	4–9 mm		
	kg	kg/m^3^	kg/m^3^	kg	kg	kg	[°C]	-
1	200	100	150.89	269.45	269.45	538.90	25	2
2	200	100	150.89	269.45	269.45	538.90	105	2
3	600	300	88.69	158.37	158.37	316.74	25	2
4	600	300	88.69	158.37	158.37	316.74	105	2
5	200	100	150.89	269.45	269.45	538.90	25	6
6	200	100	150.89	269.45	269.45	538.90	105	6
7	600	300	88.69	158.37	158.37	316.74	25	6
8	600	300	88.69	158.37	158.37	316.74	105	6
9	400	200	124.64	222.58	222.58	445.16	25	4
10	400	200	124.64	222.58	222.58	445.16	105	4
11	200	100	150.89	269.45	269.45	538.90	65	4
12	600	300	88.69	158.37	158.37	316.74	65	4
13	400	200	124.64	222.58	222.58	445.16	65	2
14	400	200	124.64	222.58	222.58	445.16	65	6

**Table 6:** Results of compressive strength test of geopolymers, MPa.

Activator [kg/m^3^]	Curing Temperature [°C]	Concentration of Alkaline Solution [mol/dm^3^]	Series No.	The Average Compressive Strength [MPa]	Standard Deviation	Variance	Coefficient of Variation [%]
Activator 100	25	2	(1)	0.33	0.0908	0.0083	27.5241
		6	(5)	0.65	0.0439	0.0019	6.69690
	65	4	(11)	1.76	0.2966	0.0880	16.8550
	105	2	(2)	3.88	0.3421	0.1170	8.81580
		6	(6)	5.12	0.4764	0.2270	9.30560
Activator 200	25	4	(9)	5.10	0.2806	0.0788	5.5024
	65	2	(13)	4.81	0.2793	0.0780	5.8063
		6	(14)	22.86	0.7127	0.5080	3.1179
	105	4	(10)	19.14	0.4278	0.1830	2.2350
Activator 300	25	2	(3)	1.23	0.0274	0.0008	2.2265
		6	(7)	5.24	0.2460	0.0605	4.6940
	65	4	(12)	10.62	0.5450	0.2970	5.1316
	105	2	(4)	11.64	0.5225	0.2730	4.4888
		6	(8)	26.92	0.5586	0.3120	2.0749

## Data Availability

Data are contained within the article.
